# The potential of games for vulnerable groups like refugees: a scoping review

**DOI:** 10.1590/1980-220X-REEUSP-2022-0365en

**Published:** 2023-10-13

**Authors:** Carla Sílvia Fernandes, Maria Joana Campos, Andreia Novo Lima, Suellen Cristina da Silva Chaves, Eliana Cristina dos Santos, Dárcio Tadeu Mendes, Maria do Perpétuo Socorro Sousa Nóbrega

**Affiliations:** 1 Escola Superior de Enfermagem do Porto, Centro de Investigação em Tecnologias e Serviços de Saúde Porto, Porto, Portugal.; 2 Escola Superior de Enfermagem do Porto, Unidade de Investigação da Escola Superior de Enfermagem do Porto, Porto, Portugal.; 3 Escola Superior de Saúde da Fundação Fernando Pessoa, Porto, Portugal.; 4 Universidade de São Paulo, Escola de Enfermagem, Departamento de Enfermagem Materno-Infantil e Psiquiátrica, São Paulo, SP, Brazil.

**Keywords:** Play and Playthings, Refugees, Gamification, Video Games, Juego e Implementos de Juego, Refugiados, Gamificación, Juegos de Vídeo, Jogos e Brinquedos, Refugiados, Gamificação, Jogos de Vídeo

## Abstract

**Objective:**

To map existing studies on the development of games for refugees, identifying the developed games, characteristics and possible application to health care.

**Method:**

A scoping review study, carried out in July 2022, using the MEDLINE^®^ (Medical Literature Analysis and Retrieval System Online), CINAHL^®^ (Cumulative Index to Nursing and Allied Health Literature), SPORTDiscus, Scopus, SciELO (Scientific Electronic Library Online), Psychology and Behavioral Sciences Collection, Cochrane Central Register of Controlled Trials databases.

**Results:**

8 studies were identified, with 8 different types of games published between 2016 and 2022. The characteristics of the games found essentially fall on their use to increase empathy towards refugees.

**Conclusion:**

This study identifies opportunities to strengthen the current body of knowledge in nursing, using games as ways of welcoming, training and integrating populations in situations of social vulnerability in which refugees find themselves.

## RESUMO

**Objetivo:**

Mapear os estudos existentes sobre o desenvolvimento de jogos para refugiados, identificando os jogos desenvolvidos, características e possível aplicação aos cuidados de saúde.

**Método:**

Estudo de revisão de escopo, realizado em julho de 2022, utilizando as bases de dados MEDLINE^®^ (*Medical Literature Analysis and Retrieval System Online*), CINAHL^®^ (*Cumulative Index to Nursing and Allied Health Literature*), SPORTDiscus, Scopus, SciELO (*Scientific Electronic Library Online*), *Psychology and Behavioral Sciences Collection*, *Cochrane Central Register of Controlled Trials*.

**Resultados:**

Foram identificados 8 estudos, com 8 diferentes tipos de jogos publicados entre 2016 e 2022. As características dos jogos encontrados recaem essencialmente sobre a sua utilização para aumentar a empatia para com os refugiados.

**Conclusão:**

Este estudo identifica oportunidades para fortalecer o atual corpo de conhecimento em enfermagem, utilizando jogos como formas de acolhimento, formação e integração de populações em situação de vulnerabilidade social em que se encontram os refugiados.

## RESUMEN

**Objetivo:**

Mapear los estudios existentes sobre el desarrollo de juegos para refugiados, identificando los juegos desarrollados, sus características y su posible aplicación a la atención de salud.

**Método:**

Estudio de revisión de alcance, realizado en julio de 2022, utilizando las bases de datos MEDLINE^®^ (Medical Literature Analysis and Retrieval System Online), CINAHL^®^ (Cumulative Index to Nursing and Allied Health Literature), SPORTDiscus, Scopus, SciELO (Scientific Electronic Library Online), Psychology and Behavioral Sciences Collection, Cochrane Central Register of Controlled Trials.

**Resultados:**

Se identificaron 8 estudios, con 8 tipos diferentes de juegos publicados entre 2016 y 2022. Las características de los juegos encontrados recaen esencialmente en su uso para aumentar la empatía hacia los refugiados.

**Conclusión:**

Este estudio identifica oportunidades para fortalecer el conocimiento actual en enfermería, utilizando el juego como forma de acoger, capacitar e integrar a poblaciones en situaciones de vulnerabilidad social en las que se encuentran refugiados.

## INTRODUCTION

There are currently millions of forcibly displaced people in the world. Among them, a large number are refugees due to civil wars in Syria, Libya, Afghanistan, Iraq and South Sudan, and, more recently, due to the war in Ukraine^(1–3)^. Indeed, refugees’ current situation in Europe is the greatest since the Second World War^(2)^. In Latin America, due to the severe economic and political instability in Venezuela, Brazil has received a high number of refugees^(4)^. All these refugee people are at risk of developing various problems.

The refugee crisis is all over the news; we hear, see and read about refugees fleeing war in search of a better place to live. Most governments have been discussing what to do with the large numbers of fugitives and how to provide them with shelter, while others are discussing how to prevent them from crossing their borders and entering their countries^(5)^.

Refugees’ needs are not determined solely by exposure to war and flight, there are life course determinants that also play a large role^(3)^. All people, including children, remain subject to levels of discrimination and social marginalization and at enormous risk of violations of their various human rights^(6)^.

The vast majority of refugees have been exposed to enormous traumas in their home country, such as human rights violations, murder of family members, imprisonment, torture and war^(1)^. In transit to their destination, they are often exposed to physical and sexual violence as well as mistreatment by traffickers and authorities^(1,6)^. But upon arrival at their destination, refugees are also exposed to post-migration stressors such as feelings of loneliness, rootlessness and social exclusion^(1,7)^. This segregation, in terms of economy and living space, is also exacerbated by the problems of social exclusion^(7)^, enhanced by the means of social communication^(8)^.

All of these factors put immense pressure on and exacerbate the already precarious mental health status of refugees who have already been subjected to trauma^(1)^. This specific group deserves special attention from health professionals, as they have different needs and vulnerabilities with regard to integration and the safeguarding of human rights^(1)^. Health professionals are ethically obligated to prepare a response to public health and health-related issues arising from the refugee movement^(2)^.

Aware of the need to intervene at this level, and as the first stage of a study in which a game will be developed to promote refugee integration into health care, we carried out this study.

It is argued that serious games can fill a gap in human rights education^(6)^, strengthening empathy skills, which is critical to the task of protecting vulnerable populations^(9)^. Due to the usefulness of digital games in promoting better socio-emotional behaviors, a lot of research has come up with interesting ways to use existing games to build socio-emotional skills^(10–14)^.

Games dealing with topics like refugees also want to convey a political message, making people to think about certain events and reflect on their own behavior^(15)^. Serious games in this field are intended to raise awareness and evoke empathy for the groups represented^(16)^. Players can feel guilt, regret, joy, pride, or shame about choices made in the game and, in the process, become aware and identify different emotions, with ethical dilemmas, opening doors to true empathy^(10)^. This aspect is important to change stereotypes and improve health professionals’ attitudes towards these vulnerable groups. This scoping review aims to map existing studies on the development of games for refugees, identifying the developed games, characteristics and possible application to health care.

## METHOD

A scoping review was carried out using the methodological framework developed by JBI Reviewer’s Manual for Scoping Reviews^(17)^ and Preferred Reporting Items for Systematic reviews and Meta-Analyses extension for Scoping Reviews (PRISMA-ScR)^(18)^. The objective, in the exploratory phase of this review, was to ensure the absence of a recent research report similar to the subject under study or a record of a review protocol. Subsequently, the research protocol was registered on the Open Science Framework^®^ platform (10.17605/OSF.IO/2VYTW).

### Search Strategy

**Chart 1:**
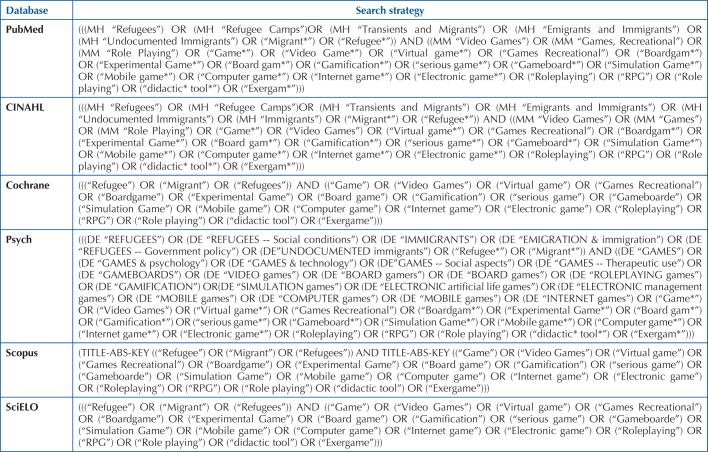
Search strategies according to database/portal. Porto, Portugal, 2022

A search was carried out in the MEDLINE® (Medical Literature Analysis and Retrieval System Online), CINAHL^®^ (Cumulative Index to Nursing and Allied Health Literature), Scopus, SciELO (Scientific Electronic Library Online), Psychology and Behavioral Sciences Collection and Cochrane Central Register of Controlled Trials databases, according to the appropriate syntax and indexing terms for each database. The search strategy is listed in Chart 1. A gray literature search was conducted using Google Scholar. A search for additional studies was also carried out in the reference lists of included articles through backward citation searching.

### Research Question Identification

This review was driven by the following questions: what games were developed for groups of refugees? What games have been developed to promote the integration of refugee groups? Which games promote greater cultural sensitivity towards refugees? What are the characteristics of games for refugees? What is its possible application to health care?

### Study Selection

The determination of inclusion and exclusion criteria was specified in accordance with the guiding questions, considering Population, Concept and Context. The following criteria were defined: in terms of Population, studies related to vulnerable groups such as refugees were included; in terms of Concept, studies related to the development of games for this population were included; and in terms of Context, all contexts were included, except those referring to refugee children’s literacy. No restrictions were made regarding the study design. From a methodological point of view, only studies with reference to some type of game assessment were included. The survey was carried out in August 2022, without time or linguistic limits. Authors of unavailable studies were contacted for clarification or additional information about their studies as suggested by the JBI methodology^(17)^.

### Data Extraction

**Figure 1:**
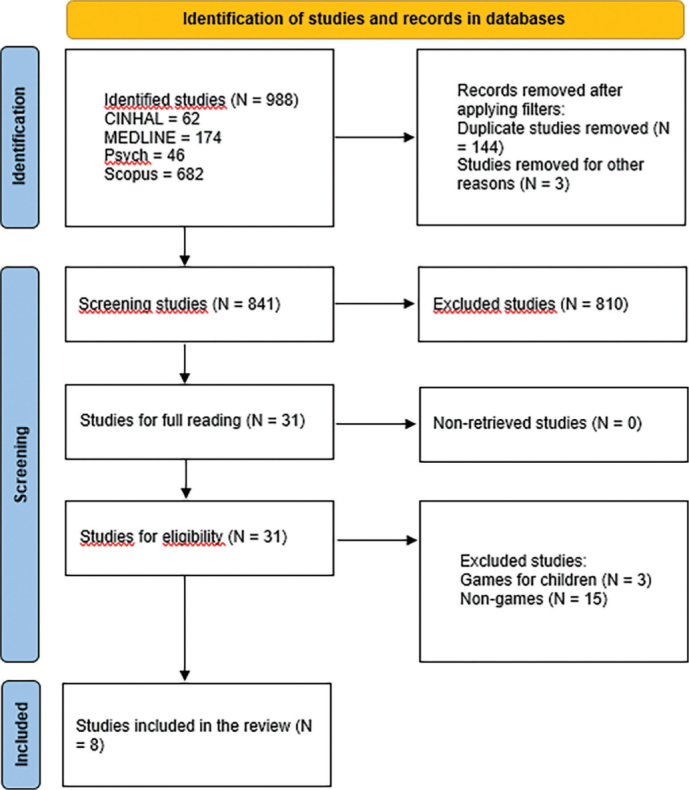
Process of article identification and inclusion – PRISMA.

For the first stages of data selection, we used the Rayyan QCRI^®^ platform (the Systematic Reviews web app). The results were assessed and selected regarding their pertinence for inclusion based on the information provided in title and abstract. Screening was performed by two authors simultaneously, and disagreements about the inclusion of studies were resolved by discussion with a third investigator. Subsequently, the selected articles were subject to a full reading that preceded their integration into the final sample. Figure 1 shows the process of identification and inclusion of articles submitted through PRISMA-ScR^(18)^

### Data Synthesis

**Chart 2:**
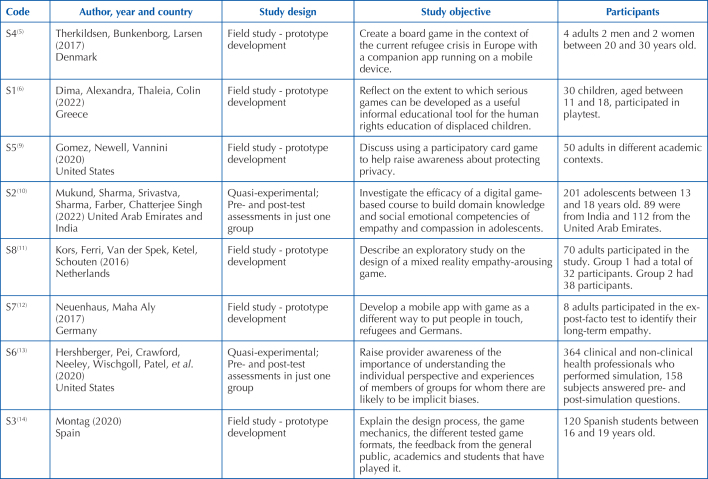
Characteristics of studies included in the scoping review. Porto, Portugal, 2022

In order to systematize the data, extracted articles were compiled descriptively with data on the place where the study was carried out, study objective, study design, participants, game title, game objective and results. In order to facilitate the presentation and discussion of results, the articles were coded, according to Chart 2. Due to the heterogeneous nature of the designs and results of the different studies integrated in the sample, only a descriptive synthesis was carried out.

## RESULTS

The search strategy retrieved 988 records. After removing duplicates, 841 records were included for the first screening and title and/or abstract analysis. After the different steps illustrated in Figure 1, 8 studies met the eligibility criteria and were included in the analysis.

### Characteristics of Included Studies

Chart 2 summarizes the characteristics of the 8 studies included in this review study with regard to authors, year, country, study design, objectives, participants, game title, game objective, ways of assessing the game and results. A total of 8 articles published between 2016 and 2022 were selected for the review^(5,6,9–14)^. The studies were developed in very different places: Greece; India; Spain; Denmark; United States (n = 2); Germany; and Netherlands. In all studies, 847 people participated in game assessment, including 5 studies with adults, 2 with adolescents, and one with children.

### Game Characteristics

**Chart 3:**
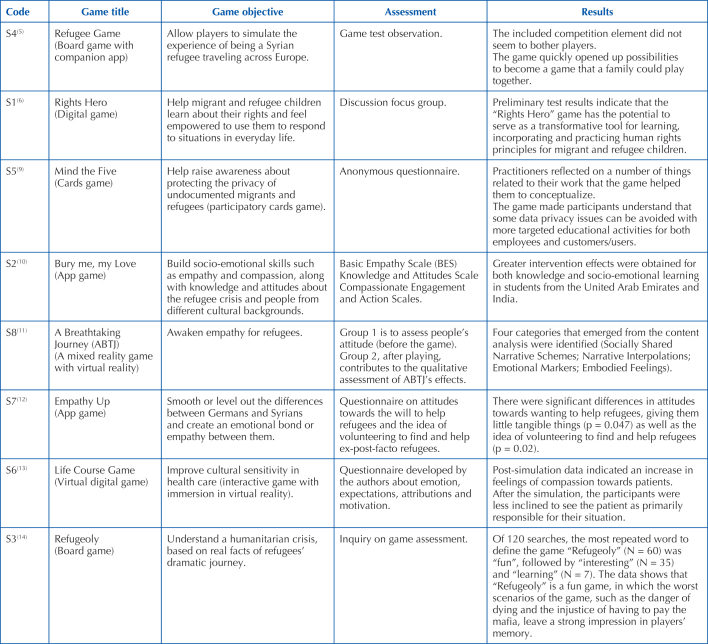
Characteristics of games included in the scoping review. Porto, Portugal, 2022

As for the type of games developed in the studies under analysis (Chart 3), two were board games^(5,14)^, one was card games^(9)^, two were for mobile phones^(10,12)^, two were digital games^(6,13)^, and one was virtual reality game^(11)^. With regard to game objectives, one of the games refers to actors’ knowledge about human rights^(6)^ and another game refers to raise awareness about refugees’ right to privacy^(9)^, but the most want to increase user empathy towards refugees^(5,10–14)^. With regard to use in health care, only one of the games was developed specifically to improve cultural sensitivity in the area of health^(13)^. The characteristics of each game are briefly described as follows.

## Game Title

### Refugee Game

“Refugee Game”^(5)^ is a board game with a companion app that addresses the Syrian refugee crisis. The aim is to allow players to simulate the experience of being a Syrian refugee traveling across Europe. Players plan a series of good choices to advance toward the game’s goal, only to discover that external events forced their decisions and made life harder or easier. The companion app is for making global changes to the game state on every playthrough, changing the values and meaning of cards already dealt so that the initial value of a card (e.g. advancing 4 spaces) is suddenly reversed^(5)^.

### The Rights Hero

“The Rights Hero”^(6)^ is a prototype digital game for children aged 11 to 18 that can be played on the computer. It was created to be used with refugee children; however, it can also be used by specialists who work with children as an educational support tool. The main objective is for superheroes to make decisions that empower them in different everyday scenarios, such as children who are not accepted at school. Players are faced with different situations that threaten to infringe on superheroes’ human rights and their ability to participate in the host community. Given this, players must choose an action from the available options. Whenever superheroes do not take proper action, thus not exercising their respective human right, their resilience is depleted, the scenario is not resolved and, in some cases, the game communicates the consequences of the wrong choice to players^(6)^.

### Mind the Five

“Mind the Five”^(9)^ is a participatory card game to help raise awareness of privacy protections for undocumented migrants or refugees. It can be used in small humanitarian organizations to promote safe and engaging information spaces for migrants and refugees. A player picks up a card of each type, and has one minute to describe an informational behavior, which corresponds to the INFO card, a type of organization, which corresponds to the ORG card, and which deserves the rating, which corresponds to the RATE card. The next player draws cards and plays the same way. After a few players have played the game, they can begin to discuss the types of information practices in their organization and how they affect vulnerable groups’ privacy^(9)^.

### Bury Me, My Love

“Bury me, my Love”^(10)^ is a mobile game whose name is a Syrian farewell phrase that roughly means: “Take care, do not even think about dying before me”. This game tells the story of a young woman who is trying to escape the war in Syria^(15,16)^. Players, however, take on the role of a husband of a woman who has to stay behind. The story unfolds through text messages, emojis and photos that the couple sends each other^(10)^. Players first encounter factors that cause sudden migration (e.g., civil war), and then are forced to guide characters through a series of ethical dilemmas. During the game, there are always references to the political situation as well as people who want to profit from the desperate situation of those who want to leave the country or information about how other countries deal with refugees^(15)^. In storytelling, players are often thrown into small decisions that reflect ethical dilemmas^(16)^.

### A Breathtaking Journey (ABTJ)

“A Breathtaking Journey”^(11)^ (ABTJ) is a mixed-reality, virtual-reality game that offers a first-person perspective of a refugee’s journey. ABTJ puts players in the shoes of a refugee who flees a war-torn country, hiding in the back of a truck, to reach a safe haven. ABTJ’s virtual experience, via goggles and sound device, is augmented with a variety of physical elements, including a mask, which utilizes a breath sensor, a scent diffuser, which mimics the interior of a truck, an unbalance motor, to simulate movement, and a controlled shutter to drop objects on players during gameplay^(11)^.

### Empathy Up

“Empathy Up”^(12)^ is a mobile game to increase empathy and try to reduce prejudices of German people against Syrian refugees. It uses a geolocation system inspired by a mixture of “Pokémon Go” and “Geo Caching”. It is like “Pokémon Go”, when moving from one place to another through a copy of a map reality, and like “Geo Caching”, which looks for certain points to allow contact with different cultural characteristics. The game allows players (Syrians and Germans) to understand more about Syrian culture, experience what refugees have gone through and finally meet each other to achieve real and direct contact^(12)^.

### Life Course Game

“Life Course Game”^(13)^ is an immersive virtual reality interactive game to improve cultural sensitivity in health care. It was originally developed and produced in 2008 by City-MatCH, and in 2017 Wright State University adapted the game to a digital and online version so that numerous medical students could play the game simultaneously. One of the game’s characters is a Syrian refugee with limited English proficiency. At age 30, the “Life Course Game” portion of simulation ends, and an immersive virtual reality (VR) scenario begins as individuals go to receive care at a community health center. VR includes four elements of the health center visit: preparation and transportation for the appointment; check-in at the health center; interaction with health professionals; and filling of prescriptions^(13)^.

### Refugeoly

“Refugeoly”^(14)^ is a board game based on the classic Monopoly model, taking on the term “Refugeoly” due to the combination of “Refugee” and “Monopoly”. It was built on refugees’ testimonies and non-governmental organization (NGO) volunteers in refugee camps in Greece, Turkey, Spain and France. The squares on the board are numbered from 0 to 39. The simply square structure helps to build, chronologically, the different situations that occur in refugees’ journey. There are three banks in the game: Player Expenses, where players put money they spend along the way; Banking is the Mafia, where most of players’ money ends up paying for a place on a boat to cross the Mediterranean, or on an illegal trip; Bank, which is an NGO. Players’ emotions keep growing as the game goes on, taking players back to the country of conflict^(14)^.

Although only one of the studies is directly applied to health care, it is observed that the games presented can promote sensitivity to refugees’ human rights, in addition to being an effective way to raise awareness about the problems they face as well as educating health professionals about the human rights of this population.

## DISCUSSION

From the perspective of developing a game to promote vulnerable group integration into health care, such as refugees, scientific evidence published in the literature on this topic was mapped. Thus, we analyzed and discussed the results in light of the questions that guided the scoping review. The first question concerns what games were developed for refugee groups. From this review, 8 studies with relatively recent studies were analyzed, between 2016 and 2022, given that the research did not delimit temporal spaces and 8 games were identified^(5,6,9–14)^.

Although the allusion to this type of game is recent, the “Escape from Woomera” game was developed in 2003, and was one of the first attempts to focus on human rights in a digital game; this and others are not documented in an article, nor do they allude to their assessment process^(15,16)^. Similar results were obtained in a systematic review study that aimed to identify the mobile applications available for refugees, where authors report that there is a limited number of articles on mobile education for refugees, most of which date from 2018, also related to the rapid increase in applications furniture from last years^(19)^.

Another issue that motivated this review was to identify which games promote the integration of groups of refugees. On this subject, only “Empathy UP” has the real intention of integrating this group. This is supposed to happen through the game’s characters, setting and story that encourages the emotional connection between the two players (locals and refugees) until they actually find themselves face to face at the end of the game^(12)^. On the other hand, “Rights Hero” promotes children to learn human rights, and its main objective is to help migrant and refugee children learn their rights and feel empowered to use them, with a view to responding to everyday life situations and indirectly, helping child integration into the communities in which they live^(6)^.

The others^(5,9–12,14)^ are games to reduce stereotypes and improve empathy, which allows us to answer the second question of this review about games that promote greater cultural sensitivity towards refugees. Hearing, seeing and feeling refugees’ stories can develop empathy in users and increase their knowledge and understanding of their lives, needs and desires^(20)^. Refugee stories function, therefore, as a second-order form of engagement with the refugees themselves and as a gesture to demonstrate trustworthiness^(20,21)^. The stories visible in the games allude to attitudes, values and judgments in order to reflect, recognize, empathize with or criticize human rights violations^(15,16)^. Analyzing these last games, it is observed that most games are immersive in which users experience the refugee role^(5,10,11,13,14)^.

Finally, with regard to its possible application to health care, only one of the studies focuses on its use in health professionals^(13)^. Indeed, the use of simulation games so far suggests that they can be initiatives that allow increasing individuals’ cultural proficiency in health professions^(13)^. The growth of the refugee population and their access to health care can lead to prejudices and negative attitudes^(22,23)^. Health professionals are no less susceptible to bias than others^(13)^. A biased and negative approach by health professionals, including nurses, can negatively affect health care quality^(22)^. The use of interactive simulation with games, in which a participant has a window into life course and experience of a patient for whom one may have negative prejudices, may result in less negative emotional responses and attitudes^(13)^. Moreover, it has been suggested that such negative attitudes can make it difficult for users to adhere to treatment, reducing the rate of use of health services and negatively affecting refugees’ physical and mental health^(22)^.

On the other hand, there is an urgent need for access and integration to health systems, given a vulnerable group that is faced with certain challenges in accessing health, including financial, administrative, language and cultural barriers as well as an insufficient understanding of how health care is organized and delivered^(24)^. It should be remembered that refugees are at greater risk of health problems, particularly mental ones, due to the high levels of trauma they have been subjected to^(4)^. These factors demonstrate a pressing need for their access and integration into health systems, especially through digital resources^(24)^, where it is possible to allocate the games. In view of this evidence, one might think that the creation and use of games both in the context of training health professionals and in accessing care can be an added value.

### Study Limitations

This path has some limitations, as only studies published with reference to game assessment were included. However, this criterion was chosen to improve quality and provide more reliable information about the games. Furthermore, one of the difficulties was the lack of specification regarding the assessment strategies of the games used in the studies.

### Contributions to Health-Related Research

This review suggests opportunities to strengthen the current body of knowledge in health and nursing, using new forms of reception, training and integration of populations in situations of social vulnerability in which refugees find themselves.

## CONCLUSION

This path allowed answering the questions that guided this research through a scoping review study with the objective of mapping existing studies on the subject, identifying the developed games, characteristics and possible application to health care. 8 studies were identified, with 8 different types of games published between 2016 and 2022. The characteristics of the games found essentially fall on their use to increase empathy towards refugees, putting players in others’ shoes, building empathy through identification and reflection. With regard to its application in health care, its use was observed in only one of the studies. However, the rest of the games allowed to address a variety of issues related to refugees, such as difficulties they face when seeking asylum, discrimination they face in their host countries, difficulties in adapting to a new culture and the need to protect their basic human rights. Moreover, games can help reduce the stigma associated with refugees and promote greater empathy and compassion in health care and by health professionals.

It is argued that, despite the important challenges, serious games can fill a gap in health professional training, but also in access to care, as more research using games is needed to determine their effectiveness.
